# Supplementary material for: Tunable thermal expansion in framework materials through redox intercalation
Source: Nat Commun. 2017 Feb 9;8:14441. doi: 10.1038/ncomms14441 (PMC5309840; doi:10.1038/ncomms14441)
Supplement: Supplementary Information — Supplementary Figures, Supplementary Tables, Supplementary Notes and Supplementary References [file ncomms14441-s1.pdf]

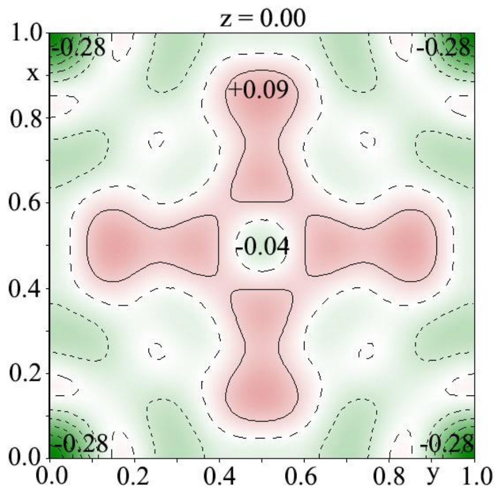

Supplementary Figure 1. Fourier neutron scattering length density difference of the (100) plane of $Li_{0.06}(Sc_{0.9}Fe_{0.1})F_3$ assuming the lithium ions at the A-site at room temperature. Although the content of lithium is small, the fitting result is significantly improved upon adding the lithium ions at the A-site position (The values of R(obs) = 1.27%, WR(obs) = 1.78% are much reduced to R(obs) = 1.07%, WR(obs) = 1.53%, respectively).

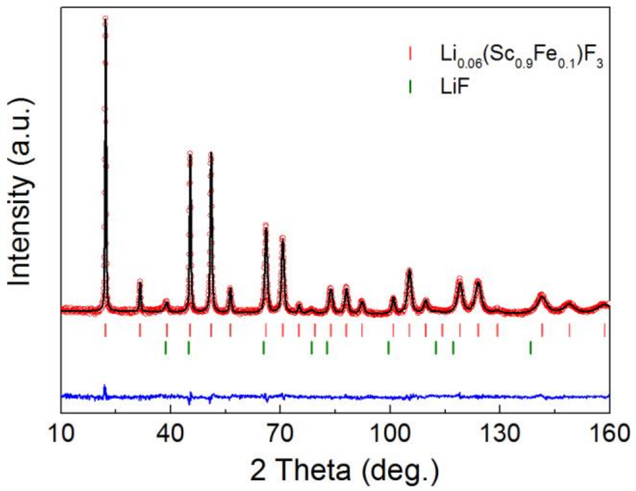

Supplementary Figure 2. Neutron powder diffraction refinement (λ = 1.5398 Å) for $Li_{0.06}(Sc_{0.9}Fe_{0.1})F_3$ at 298 K. There is a very small amount (0.8 wt%) of impurity phase LiF.

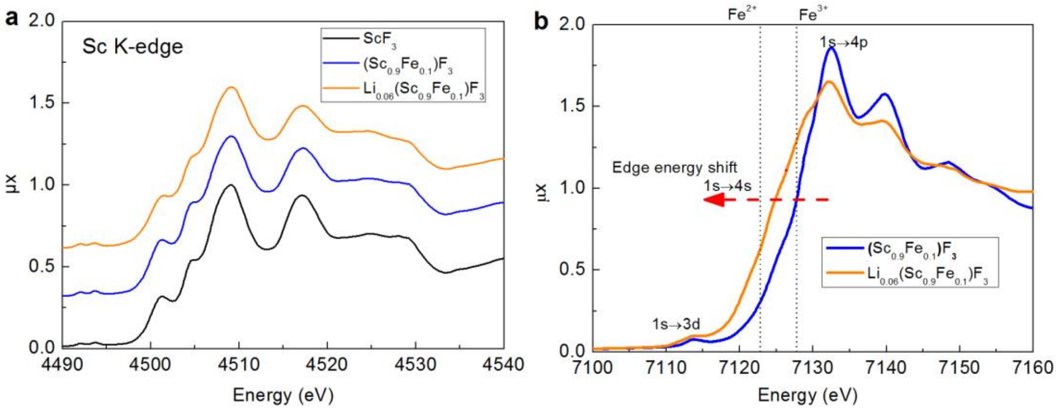

Supplementary Figure 3. The Sc K-edge and Fe K-edge XANES of ScF_3, $(Sc_{0.9}Fe_{0.1})F_3$ and $Li_{0.06}(Sc_{0.9}Fe_{0.1})F_3$ at room temperature. (**a**) The Sc K-edge XANES. It can be seen that the change around the Sc K-edge is negligible after lithiation, indicating Sc valence does not change. (**b**) The Fe K-edge XANES of $(Sc_{0.9}Fe_{0.1})F_3$ and $Li_{0.06}(Sc_{0.9}Fe_{0.1})F_3$. The decrease of Fe valence state is confirmed in $Li_{0.06}(Sc_{0.9}Fe_{0.1})F_3$ according to the shift of the absorption edge to the lower energy.

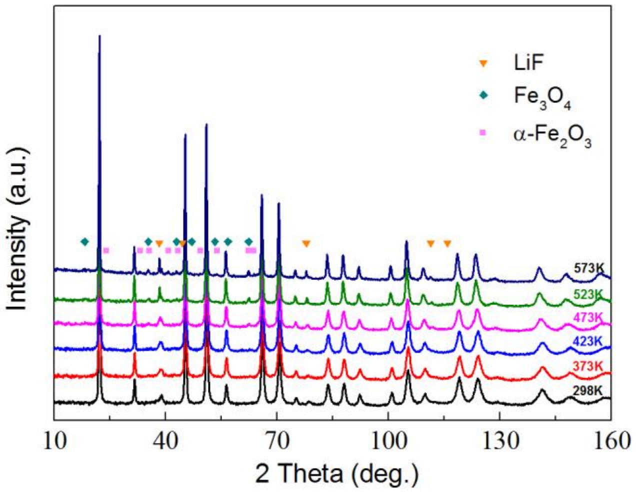

Supplementary Figure 4. Neutron powder diffraction of the $Li_{0.06}(Sc_{0.9}Fe_{0.1})F_3$ sample as function of temperature for the first thermal treatment. With increasing temperature, the ions of Li and Fe gradually separate from the lattice of $Li_{0.06}(Sc_{0.9}Fe_{0.1})F_3$ to form impurity phases of LiF, Fe_3O_4, and α-Fe_2O_3.

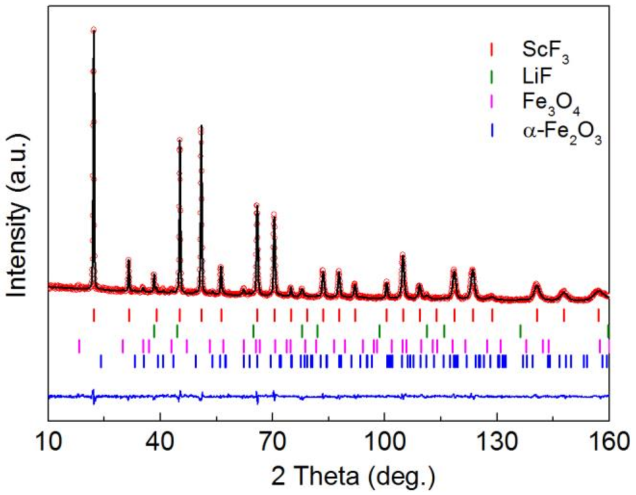

Supplementary Figure 5. The structure refinement using neutron powder diffraction of the $Li_{0.06}(Sc_{0.9}Fe_{0.1})F_3$ sample at 573 K. The main ReO_3-type phase is ScF_3, and the other impurity phases are LiF, Fe_3O_4, and α-Fe_2O_3.

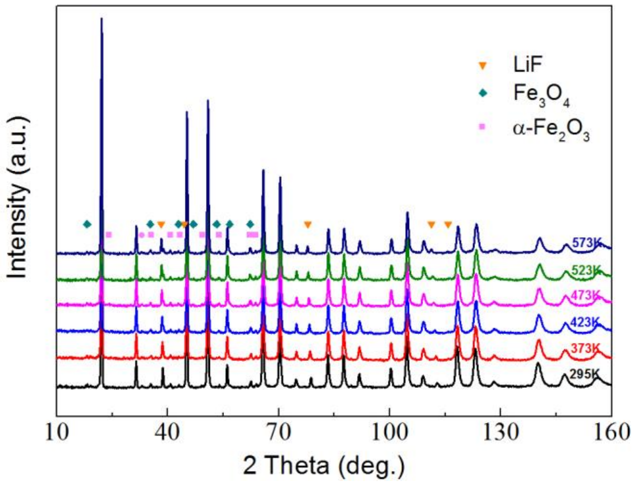

Supplementary Figure 6. Neutron powder diffraction of the $Li_{0.06}(Sc_{0.9}Fe_{0.1})F_3$ sample as function of temperature for the second thermal treatment. Here the $Li_{0.06}(Sc_{0.9}Fe_{0.1})F_3$ sample was heated again after the first thermal treatment to collect the temperature dependence of neutron powder diffraction data. The phase content of ScF_3, LiF, Fe_3O_4, and α-Fe_2O_3 does not change as function of temperature for the 2nd run.

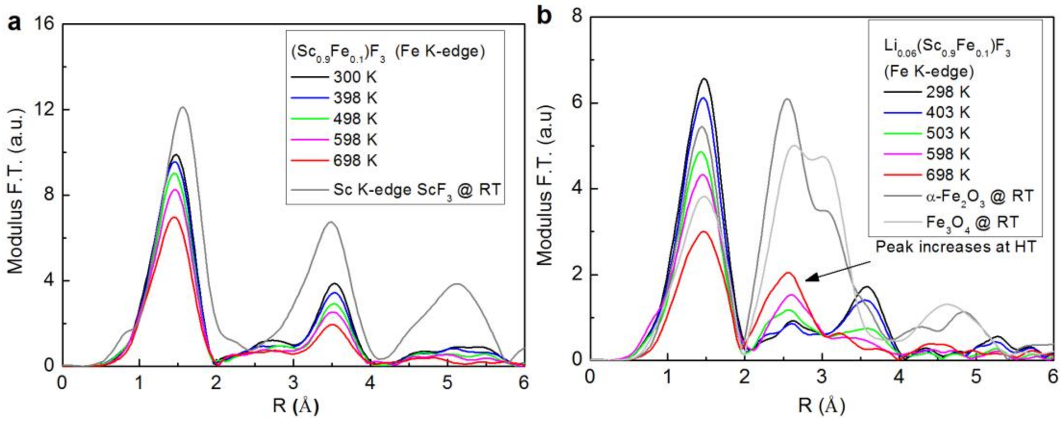

Supplementary Figure 7. Temperature dependence of the Fourier transform of the Fe K-edge $k^3\chi(k)$ EXAFS signal measured in the $(Sc_{0.9}Fe_{0.1})F_3$ and $Li_{0.06}$ $(Sc_{0.9}Fe_{0.1})F_3$ samples (left and right panels, respectively). As a reference, we show the FT of the Sc K-edge EXAFS of pure ScF_3 in **a,** and of the Fe K-edge EXAFS of α-Fe_2O_3 and Fe_3O_4 oxides in **b**. The $k^3\chi(k)$ weighted EXAFS signals were Fourier transformed in the interval k = 2–14 $Å^{-1}$ using a Gaussian window.

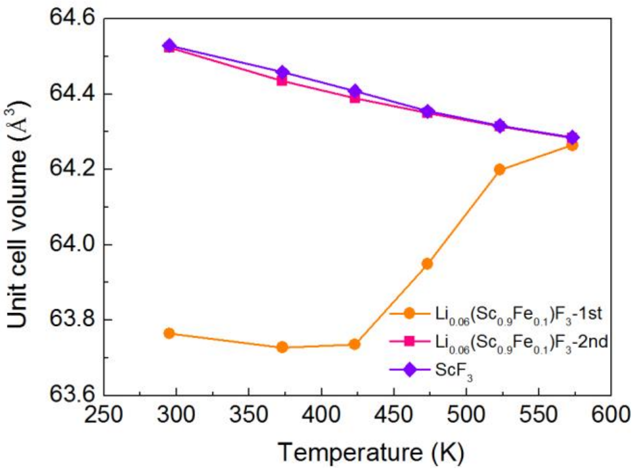

Supplementary Figure 8. Temperature dependence of unit cell volume of the main ReO_3-type phase of the $Li_{0.06}(Sc_{0.9}$ $Fe_{0.1})F_3$ sample for the first and second thermal treatment by means of neutron powder diffraction. For the 1st run, the large increase in the unit cell volume is resulted from the gradual delithiation process from $Li_{0.06}(Sc_{0.9}$ $Fe_{0.1})F_3$ to ScF_3. However, for the 2nd run the unit cell volume almost overlaps with those of ScF_3 due to the fact that the main ReO_3-type phase of the $Li_{0.06}(Sc_{0.9}Fe_{0.1})F_3$ sample transforms to ScF_3 after annealing at 573 K.

Supplementary Table 1. The agreement factors of Rietveld refinement of neutron powder diffraction of $Li_{0.06}(Sc_{0.9}Fe_{0.1})F_3$ at 298 K. The refinements were tried for the three typical compositions assuming Li ions at the A-site. If assuming lithium ions at the B-site the refinement became much worse. The best refinement obtained the composition of $Li_{0.06}(Sc_{0.9}Fe_{0.1})F_3$ in which the lithium ions are at the A-site.

Composition			R_p (%)	R_{wp} (%)	χ^2
Sc	Fe	Li			
0.9	0.1	0	3.43	4.38	1.27
0.9	0.1	0.1	3.48	4.41	1.29
0.9	0.1	0.06	3.41	4.34	1.25

Supplementary Table 2. The agreement factors of Rietveld refinement of neutron powder diffraction of the $Li_{0.06}(Sc_{0.9}Fe_{0.1})F_3$ sample at 573 K. The best refinement is that the main ReO_3-type phase of the $Li_{0.06}(Sc_{0.9}Fe_{0.1})F_3$ sample is ScF_3, indicating that the sample actually transforms to ScF_3. The ions of Li and Fe exist as other phases of LiF and Fe oxides, respectively.

Composition			R_p (%)	R_{wp} (%)	χ^2
Sc	Fe	Li			
1	0	0	3.50	4.41	1.33
0.95	0.05	0	3.51	4.43	1.35
0.9	0.1	0	3.54	4.47	1.37
0.9	0.1	0.06	3.55	4.47	1.37
0.9	0.1	0.03	3.53	4.45	1.36

Supplementary Table 3. The phase content (wt%) of SFF and LSFF samples.

Sample	ReO_3-type phase	LiF	Fe_3O_4	Fe_2O_3
$(Sc_{0.9}Fe_{0.1})F_3$ (SFF)	100%	-	-	-
$Li_{0.06}(Sc_{0.9}Fe_{0.1})F_3$ (LSFF-1)	99.2%	0.8%	-	-
$Li_{0.04}(Sc_{0.94}Fe_{0.06})F_3$ (LSFF-2)	95.9%	1.2%	2.9%	-
$Li_{0.02}(Sc_{0.97}Fe_{0.03})F_3$ (LSFF-3)	93.4%	1.6%	4.2%	0.8%
ScF_3 (LSFF-4)	90.8%	2.0%	6.0%	1.2%

Supplementary Table 4. Anisotropic atomic displacement parameters of F ions and CTEs of LSFF samples. U_{33} and U_{11} are atomic displacement parameters of F ions for the transverse and longitudinal directions, respectively.

Samples	U_{11} (Å^2)	U_{33} (Å^2)	U_{33}/U_{11}	CTE ($10^{-6}K^{-1}$)
$Li_{0.06}(Sc_{0.9}Fe_{0.1})F_3$ (LSFF-1)	0.0077(2)	0.0287(2)	3.7	1.03
$Li_{0.04}(Sc_{0.94}Fe_{0.06})F_3$ (LSFF-2)	0.0075(2)	0.033(1)	4.4	-0.75
$Li_{0.02}(Sc_{0.97}Fe_{0.03})F_3$ (LSFF-3)	0.0065(1)	0.0369(1)	5.7	-2.59
ScF_3 (LSFF-4)	0.005(1)	0.0469(2)	9.4	-7.40

Supplementary Note 1. Structure and chemical composition characterisation of $Li_{0.06}(Sc_{0.9}Fe_{0.1})F_3$.

The structure and chemical composition of $Li_{0.06}(Sc_{0.9}Fe_{0.1})F_3$ was mainly determined by neutron powder diffraction (NPD). The cubic structure model ($Pm\bar{3}m$) was adopted for structure refinement. Li, Sc/Fe, and F atoms are at 1b, 1a, and 3d sites, respectively. Firstly, neutron scattering Fourier difference maps between the observed and calculated structure factors were obtained refining against NPD data for the structural determination of the Li ions environment (Fig. 2b in the main text). The calculated structure factors were obtained according to the structural model without any Li ion. The negative peak at the (0,0,0) indicates the position of Li ions, since Li has a negative neutron scattering length (b = -1.90 fm)[1]. Indeed, the refinement is highly improved by assuming that Li ions are located at the (0,0,0) (Supplementary Fig. 1). We can obtain that Li ions are inserted into the A-site of the cubic cell formed by ScF_6 octahedra (Fig. 1a in the main text). Secondly, the stoichiometry of Li in $Li_x(Sc_{0.9}Fe_{0.1})F_3$ was determined by refining occupancy of Li. Various x value was tried in the range of $0.0 \leq x \leq 0.1$. The best refinement result revealed that Li occupancy is 0.06 at the A-site. The occupancies of Sc and Fe were also tried to be refined, giving that the best result for the stoichiometry of Sc and Fe is to be the nominal ones from the precursor of $(Sc_{0.9}Fe_{0.1})F_3$. The refinement results are shown in Supplementary Fig. 2 and Table 1. Atomic displacement parameters are assumed to be isotropic for Li, Sc/Fe atoms, but anisotropic for F atoms (U_{11} and U_{33}).

The wet-chemical experiments of inductively coupled plasma spectroscopy (ICP) analysis were also carried out to determine the composition. For the sample of LSFF-1, the measured atomic ratio of Sc : Fe : Li is 1.0 : 0.11 : 0.12. After deducting the contribution from the impurity phase of LiF (0.8 wt%), the chemical composition would be $Li_{0.065}(Sc_{0.9}Fe_{0.099})F_3$ with the normalization of Sc stoichiometry to 0.9. There is a good agreement with the results calculated by NPD data.

Supplementary Note 2. Characterisation of chemical valence of Sc and Fe in the ScF_3-based compounds.

The X-ray absorption near edge structure (XANES) spectroscopy is sensitive to the electronic and geometrical structure around the atom of interest, and the edge position of the spectra is indicative of its oxidation state. The pre-edge peak (see Fig. 2f-h in the main text), corresponding to the 1s→3d transition, can be used to estimate the $Fe^{3+}/\sum Fe$ ratio for $Li_{0.06}(Sc_{0.9}Fe_{0.1})F_3$, since there is a well established correlation between the energy of the pre-edge and the $Fe^{3+}/\sum Fe$ ratio[2,3]. When the $Fe^{3+}/\sum Fe$ ratio decreases, the energy of the 1s→3d pre-edge centroid shifts to lower energy. After the background subtraction, one can clearly see that the pre-edge centroid shifts to lower energy (Fig. 2h in the main text). For $(Sc_{0.9}Fe_{0.1})F_3$, the $Fe^{3+}/\sum Fe$ ratio is near one what means that Fe ions are at the valence 3+. However for $Li_{0.06}(Sc_{0.9}Fe_{0.1})F_3$, the $Fe^{3+}/\sum Fe$ ratio is about 0.36, which means that a large portion of Fe ions were reduced from Fe^{3+} to Fe^{2+}. The results of Fe K-edge XANES agree with the composition of $Li_{0.06}(Sc_{0.9}Fe_{0.1})F_3$ obtained by NPD refinement.

Supplementary Note 3. Phase transformation of $Li_{0.06}(Sc_{0.9}Fe_{0.1})F_3$ as function temperature.

In order to understand the effects of high temperature on chemical stability, we measured the temperature dependence of NPD for the $Li_{0.06}(Sc_{0.9}Fe_{0.1})F_3$ sample. With increasing temperature, the delithiation process takes place and impurity phases of LiF, Fe_3O_4, and α-Fe_2O_3 gradually increases (Supplementary Fig. 4 and Table 3). That means that Fe and Li ions separate from the lattice of the ReO_3-type phase to form those

impurities. The main ReO_3-type phase has progressively lower concentrations of Li and Fe ions for the higher annealing temperatures. Simultaneously, the lattice constant of the main ReO_3-type phase increases (Supplementary Fig. 8). The chemical composition of the annealed $Li_{0.06}(Sc_{0.9}Fe_{0.1})F_3$ sample at various temperatures can be estimated according to the phase content by means of full-profile Rietveld refinements, which is provided in Fig. 3b in the main text. If annealed at temperatures as high as 573 K, the lattice constant of the main ReO_3-type phase is 4.005Å, which is almost identical to that of ScF_3 (4.006Å at 573 K). This suggests that all Fe and Li ions are separated from the lattice of ScF_3, and thus the main ReO_3-type phase becomes pure ScF_3. This is verified by the high temperature NPD measurement of the $Li_{0.06}(Sc_{0.9}Fe_{0.1})F_3$ sample after decomposition showing that, the main ReO_3-type phase exhibits the same NTE property with that of ScF_3 (Supplementary Fig. 8).

The phase evolution of $Li_{0.06}(Sc_{0.9}Fe_{0.1})F_3$ as a function of temperature can be further observed by extended X-ray absorption fine structure (EXAFS) spectroscopy, since this technique provides structural information as coordination numbers and interatomic distances around the absorbing element. The temperature dependence of Fourier transform of the weighted $k^3\chi(k)$ EXAFS signal at the Fe K-edge for both $Li_{0.06}(Sc_{0.9}Fe_{0.1})F_3$ and $(Sc_{0.9}Fe_{0.1})F_3$ is displayed at Supplementary Fig. 8. For comparison, the spectra collected at the Sc K-edge are also included, as well as the references α-Fe_2O_3 and Fe_3O_4 at the Fe K-edge. For the local structure of Fe in $Li_{0.06}(Sc_{0.9}Fe_{0.1})F$, the first peak, corresponding to the Fe-F bonds, decreases, and the second peak at 2.5 Å (no phase correction) rises with increasing temperature. It is in good agreement with the formation of Fe oxides which is already hinted by NPD (Supplementary Fig. 4 and Table 3). On the other hand, for the $(Sc_{0.9}Fe_{0.1})F_3$ sample it is only found the expected damping of the peaks due to the increasing thermal disorder. The local structure does not change and is similar to that of Sc atoms in ScF_3. This shows that Fe ions are stable in the lattice of $(Sc_{0.9}Fe_{0.1})F_3$ on heating but not for $Li_{0.06}(Sc_{0.9}Fe_{0.1})F_3$.

Supplementary References

1. Pérez-Estébanez, M., Isasi-Marín, J., Többens, D. M., Rivera-Calzada, A. & León, C. A systematic study of Nasicon-type $Li_{1+x}M_xTi_{2-x}(PO_4)_3$ (*M*: Cr, Al, Fe) by neutron diffraction and impedance spectroscopy. *Solid State Ionics* **266,** 1–8 (2014).

2. Berry, A. J. *et al.* XANES calibrations for the oxidation state of iron in a silicate glass. *Am. Mineral*. **88,** 967–977 (2003).

3. Wilke, M. *et al.* The oxidation state of iron determined by Fe K-edge XANES - application to iron gall ink in historical manuscripts. *J. Anal. At. Spectrom*. **24**, 1364–1372 (2009).
